# Clinical outcomes and end-of-life treatment in 596 patients with isolated traumatic brain injury: a retrospective comparison of two Dutch level-I trauma centers

**DOI:** 10.1007/s00068-023-02407-5

**Published:** 2024-01-16

**Authors:** Menco J. S. Niemeyer, Denise Jochems, Jan C. Van Ditshuizen, Janneke de Kanter, Lotte Cremers, Martijn van Hattem, Dennis Den Hartog, Roderick Marijn Houwert, Luke P. H. Leenen, Karlijn J. P. van Wessem

**Affiliations:** 1 Department of Trauma Surgery, University Medical Center Utrecht, Heidelberglaan 100, 3584 CX Utrecht, The Netherlands; 2 Trauma Research Unit, Department of Surgery, Erasmus MC, University Medical Center Rotterdam, Rotterdam, The Netherlands; 3 Trauma Center Southwest Netherlands, Erasmus MC, University Medical Center Rotterdam, Rotterdam, The Netherlands; 4 Department of Radiology, UMC Division Imaging and Oncology, University Medical Center Utrecht, Utrecht, The Netherlands; 5 Department of Radiology, Erasmus MC, University Medical Center Rotterdam, Rotterdam, The Netherlands; 6 Department of Radiology, Jeroen Bosch Hospital, ‘s Hertogenbosch, The Netherlands

**Keywords:** Traumatic brain injury, In-hospital mortality, Withdrawal of life sustaining treatment, End of life, ICU

## Abstract

**Purpose:**

With an increasingly older population and rise in incidence of traumatic brain injury (TBI), end-of-life decisions have become frequent. This study investigated the rate of withdrawal of life sustaining treatment (WLST) and compared treatment outcomes in patients with isolated TBI in two Dutch level-I trauma centers.

**Methods:**

From 2011 to 2016, a retrospective cohort study of patients aged ≥ 18 years with isolated moderate-to-severe TBI (Abbreviated Injury Scale (AIS) head ≥ 3) was conducted at the University Medical Center Rotterdam (UMC-R) and the University Medical Center Utrecht (UMC-U). Demographics, radiologic injury characteristics, clinical outcomes, and functional outcomes at 3–6 months post-discharge were collected.

**Results:**

The study population included 596 patients (UMC-R: *n* = 326; UMC-U: *n* = 270). There were no statistical differences in age, gender, mechanism of injury, and radiologic parameters between both institutes. UMC-R patients had a higher AIShead (UMC-R: 5 [4–5] vs. UMC-U: 4 [4–5], *p* < 0.001). There was no difference in the prehospital Glasgow Coma Scale (GCS). However, UMC-R patients had lower GCSs in the Emergency Department and used more prehospital sedation. Total in-hospital mortality was 29% (*n* = 170), of which 71% (*n* = 123) occurred after WLST. Two percent (*n* = 10) remained in unresponsive wakefulness syndrome (UWS) state during follow-up.

**Discussion:**

This study demonstrated a high WLST rate among deceased patients with isolated TBI. Demographics and outcomes were similar for both centers even though AIShead was significantly higher in UMC-R patients. Possibly, prehospital sedation might have influenced AIS coding. Few patients persisted in UWS. Further research is needed on WLST patients in a broader spectrum of ethics, culture, and complex medical profiles, as it is a growing practice in modern critical care.

**Level of evidence:**

Level III, retrospective cohort study.

## Introduction

Mortality due to traumatic brain injury (TBI) in the Intensive Care Unit (ICU) is rising globally. A shift has been observed in causes of trauma related deaths from multi organ failure and exsanguination to deaths related to the central nervous system [1]. Advancements in resuscitation and acute care strategies have attributed to hemodynamically unstable and severe polytraumatized patients surviving the acute phase more often with TBI becoming the major determinant for ICU mortality [2–4].

Survival of moderate-to-severe TBI can result in a spectrum of morbidities from mild disabilities allowing independency in daily functioning, to severe conditions such as unresponsive wakefulness syndrome (UWS) [5]. In a previous retrospective study among 179 isolated TBI patients, approximately 30% of patients remained unable to live independently after 4 months [4].

Outcome in patients with TBI is difficult to predict, despite several attempts at developing prediction models. The Abbreviated Injury Scale (AIS) and derived Injury Severity Scores (ISS) are both widely verified and applied entities for hospital benchmarking and research [6, 7]. However, they prove unreliable when applied in differing trauma systems [8]. In addition, the most well-known brain injury-prediction models (‘IMPACT’ and ‘CRASH’) attempt prognostication for research populations but lack clinical feasibility [9, 10]. These models only discriminate between dead, favorable, and unfavorable outcome. In addition, when physicians decide to withdraw life sustaining treatment (WLST) based on the factors of these prognostic models, they may inadvertently confirm the model’s predictions with a risk of self-fulfilling prophecies that may occur with WLST. This risk has been recognized for patients with different kinds of acute brain injury [11, 12]. This limitation—among others—leaves the surgical team without an evidence-based framework for acute-phase decision-making combining outcome prediction and ethics in treatment [9].

Traumatic brain injury seldom comes expected. Therefore, decisions following resuscitation and stabilization must be made in consultation with next of kin rather than by the unconscious patient in case a patient has no recorded advance directives. When a patient’s prognosis is deemed medically futile, or next of kin argue the patient would have never wanted the predicted outcome, physicians can decide to withhold or withdraw life sustaining treatment. In-hospital WLST rates in patients with TBI range from 45 to 87% [2, 13, 14]. Unfortunately, comparative literature is scarce. Furthermore, there is considerable debate regarding differences in WLST rates following TBI, suggesting these are influenced culturally, by specific hospital practices, and even by the individual surgeon-on-call [15, 16].

The Netherlands is one of the first countries in the world to adopt end-of-life legislature [17]. In turn, Dutch WLST practice has been received ambiguously in comparative literature. A study by Wade et al. on prolonged consciousness disorders even excluded Dutch data from analysis, which exemplifies the point of view on Dutch practice; “*Studies from the Netherlands all emphasize the low prevalence there, attributed to their particular clinical practice*.”[18]. This view on the Dutch practice was confirmed by data from a recent single-center study in which life sustaining therapy was withdrawn in 82% of deceased TBI patients [2].

This study aimed to investigate outcomes, and WLST rates in patients with isolated moderate-to-severe TBI in two comparable major Dutch level-1 trauma centers with 24/7 neurosurgical care were investigated. It was hypothesized that there would be no differences in outcome between these two centers.

## Methods

This research has been performed in concurrence with the Strengthening the Reporting of Observational studies in Epidemiology (STROBE) guidelines [19].

### Setting

Erasmus MC, University Medical Center in Rotterdam (UMC-R) is the largest level-I trauma center in the Netherlands servicing 2.2 million residents in an area of 3,500 square kilometers with an annual admission rate around 1500 trauma patients. It is characterized by industry and port facilities, highly urbanized, and services the archipelagic province of Zeeland as well [20]. University Medical Center Utrecht (UMC-U) services the central province of the Netherlands with a relatively smaller but more densely populated area of 1,500 square kilometers and approximately 1.3 million residents. Around 1300 trauma patients with full activation of a trauma team are annually admitted [21].

### Study design and data collection

A retrospective cohort study was conducted from January 2011 until December 2016 in two Dutch level-I trauma centers. Patients were selected from the respective regional trauma registration of both UMC-R (Trauma center Southwest Netherlands) and UMC-U (Trauma center mid-Netherlands). Clinical and outcome data were extracted manually by clinician researchers from medical files from both centers. Both AIS and ISS were assessed and calculated by trained coders in their respective trauma registration. Previous research has shown variable accuracy of coding AIS of the head [8]. To assess accuracy and correction for inter-observer variability, coders from both trauma centers cross-checked all AIS codes. The intraclass correlation coefficient was used for post hoc analysis of the AIS scores and interpreted according to the index by Landis and Koch ranging from Slight (0.0–0.02) to almost perfect (0.81–1.0), also displayed in Appendix Table 4 [22].

### Patient sample

Patients with moderate-to-severe TBI were included if they were admitted to ICU in UMC-U or UMC-R from January 2011 until December 2016. Moderate–severe isolated TBI was defined as AIShead ≥ 3 and AIS ≤ 2 in other anatomic regions. Patients aged ≥ 16, patients with isolated cervical spinal injury, and patients who had been transferred between hospitals were excluded. Subdural and/or parenchymal hemorrhages in patients without evident trauma, on imaging or clinically, were considered as non-traumatic hemorrhages and these patients were excluded.

### Clinical variables

The trauma registries of both centers provided baseline characteristics including demographics (age and gender) and injury characteristics. These included ISS ‘98, AIS’98, Helicopter-assisted Emergency Medical Service (HEMS) involvement, prehospital Glasgow Coma Scales (GCS), Revised Trauma Scores (RTS), and associated Probability of Survival (PS) [23–25]. Other admission parameters were collected from patient records, including trauma mechanism, ethanol levels, Glasgow Coma Scale (GCS), and relevant stem reflexes assessed by the neurologist-on-call. The use of anticoagulants was registered, and the Charlson Comorbidity Index (CCI) score was calculated and used to adjust for confounding by comorbidity [26]. Data on neurosurgical interventions included intracranial pressure (ICP) monitor placement and decompressive craniotomies.

### Imaging variables

All acute-phase computed tomography imaging in both centers were reassessed by two sets of two neuro-radiologist who were blinded for patient outcome. A format based on the Rotterdam system for grading brain imaging was used [27]. Reassessments were scored for the presence of compression of the basal cisterns or third ventricle, midline shift > 5 mm, and the frequency of epidural, subdural, and/or subarachnoid hemorrhage.

### Outcome variables

Outcomes observed included rate of WLST, lengths of stay (LOS) both in ICU and hospital, in-hospital mortality, cause of death, and functional outcome data during follow-up (i.e., Glasgow Outcome Scale including mortality during follow-up). Rates of WLST were assessed based on patient records. WLST was defined as discontinuation of treatment in hemodynamically supported yet stable patients. Patients who were confirmed brain dead, who were suspected brain dead on arrival, or died due to other causes were excluded from WLST subset analysis. Cause of death was extracted from patient records. Functional outcome measured by Glasgow Outcome Scale (GOS) was collected after 3 months from records of outpatient clinic visits, or correspondence from a rehabilitation hospital [5]. In case of missing data at 3 months, the first available GOS within 6 months was used. If this was before 3 months’ time but lost to follow-up afterward, it was recorded as 3-month follow-up data since further deterioration was not expected. In case patients were lost to follow-up, mortality could still be registered by consulting the Dutch national registry of deceased persons.

### Statistical analysis

All statistical analyses were performed using IBM SPSS Statistics, version 21.0.0 (Armonk, NY, USA). Group differences between survivors and patients who died due to WLST, in addition to group differences in-hospital mortality, GOS, and frequency of WLST between the UMC-U and UMC-R, were calculated using a Mann–Whitney U test in case of continuous, non-normally distributed, variables. In case of a different shape of distributions in each group, mean ranks were compared between groups, and medians are shown. Differences in distribution of categorical or ordinal variables between groups were calculated with the Chi-square test for homogeneity. Fisher’s exact test instead of a Chi-square test was used if the expected cell count was less than five. For analysis of inter-observer variability, a two-way mixed, single-measures intraclass correlation coefficient (ICC) was used for ordinal variables. Statistical significance was defined as *p* < 0*.*05, where possible frequencies and percentages are given. Continuous variables are displayed as median [Q1–Q3].

## Results

**Fig. 1:**
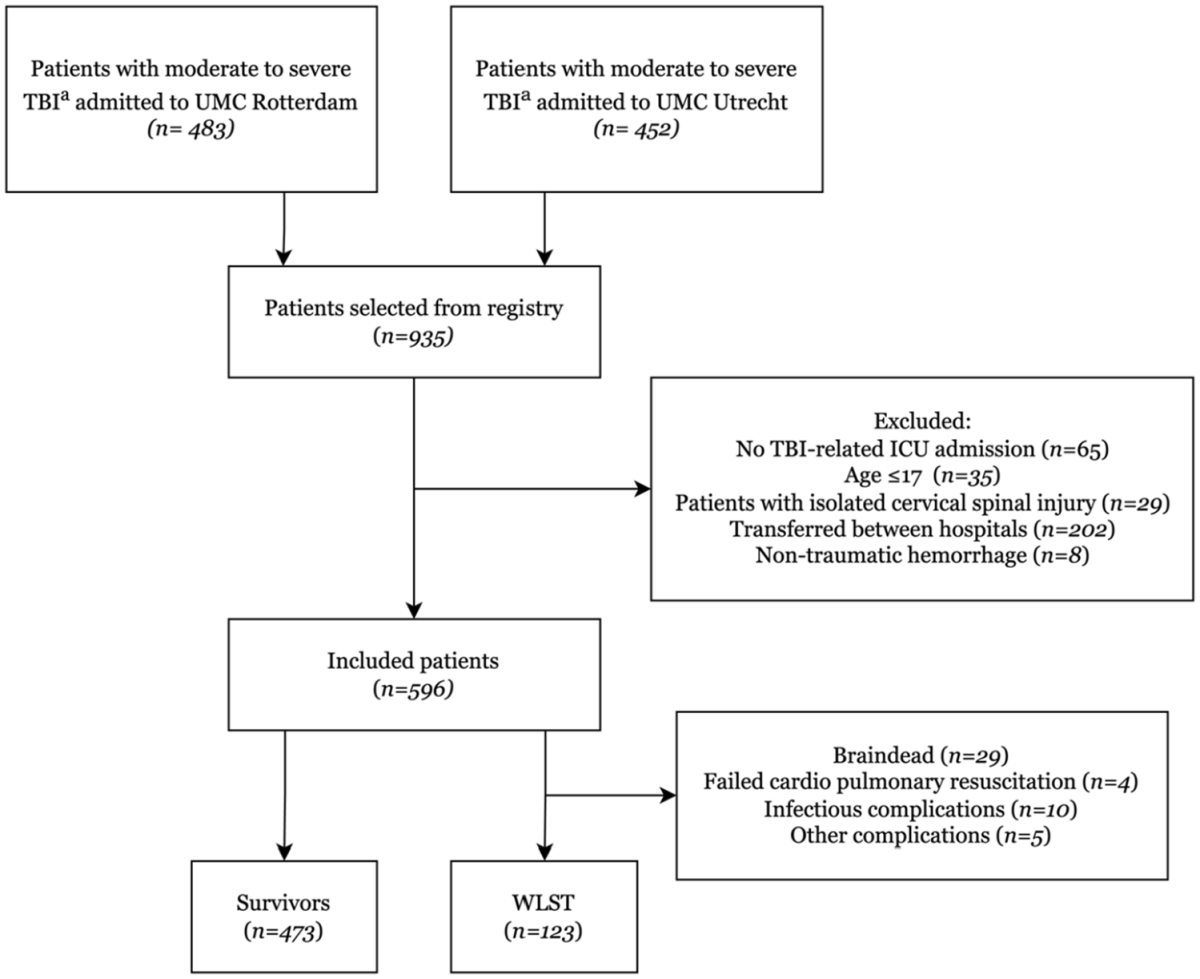
Flowchart of patient selection. **a**: Defined as AIShead ≥ 3 and AIS in other anatomic regions ≤ 2. *TBI* traumatic brain injury, *UMC* University Medical Center, *ICU* Intensive Care Unit, *WLST* withdrawal of life sustaining treatment

A total of 596 patients were included: 326 patients from UMC-R and 270 patients from UMC-U. A flowchart of patient inclusion is presented in Fig. 1. The majority of patients in both centers was male (66 vs. 61%, *p* = 0.253). In addition, there was no difference in age (median of 57 years in both groups, *p* = 0.714) and Charlson Comorbidity Index (median 0 in both groups, *p* = 0.230). Most patients in both groups were involved in traffic accidents (UMC-R: *n* = 130, 40%; UMC-U: *n* = 127, 47%), followed by falls from height (UMC-R: *n* = 106, 33%; UMC-U: *n* = 74, 27%) (Table 1). Patients from both centers had comparable Revised Trauma Score (*p* = 0.876), and subsequent Probability of Survival (*p* = 0.275). However, AIShead scores in patients from UMC-R were statistically higher (UMC-R: 5 [4, 5] vs. UMC-U: 4 [4, 5], *p* < 0.001) and had statistically higher ISS compared to UMC-U patients (UMC-R patients had ISS of 25 [20–26] vs. ISS of 21 [17–26] in UMCU-U patients, *p* < 0.001, Table 1). Post hoc AIShead analysis showed fair agreement (ICC 0.356) among coders in UMC-U patients, and moderate agreement (ICC 0.534) in UMC-R patients.

**Table 1:** Baseline, injury, and radiologic variables

Variable	Total population *n* = 596	UMC-R *n* = 326	UMC-U *n* = 270	*p* value
Demographics				
Male gender	379 (64)	214 (66)	165 (61)	0.253
Age	57 [38–70]	57 [38–71]	57 [38–70]	0.714
Charlson comorbidity index	0 [0–1]	0 [0–1]	0 [0–1]	0.230
Injury characteristics				
Mechanism of injury				0.793
Fall low height,	101 (17)	50 (16)	51 (18)	
Fall from height	180 (30)	106 (33)	74 (27)	
Road traffic accidents	257 (43)	130 (40)	127 (47)	
Penetrating injuries	23 (4)	18 (6)	5 (2)	
Other	35 (6)	22 (7)	13 (5)	
RTS	5.030 [4.094–6.904]	5.030 [4.094–6.904]	5.030 [4.094–6.904]	0.876
Probability of Survival^a^	0.77 [0.50–0.91]	0.75 [0.47–0.90]	0.79 [0.46–0.93]	0.275
ISS	24 [17–26]	25 [20–26]	21 [17–26]	< 0.001*
AIS head	4 [4, 5]	5 [4, 5]	4 [4, 5]	0.003*
AIS face	0 [0–2]	0 [0–2]	0 [0–2]	< 0.001*
Use of HEMS	308 (52)	214 (66)	94 (35)	< 0.001*
Prehospital GCS	7 [3–12]	7 [3–11]	7 [3–12]	0.626
Use of prehospital sedation	382 (64)	273 (84)	109 (40)	< 0.001*
GCS in ED	6 [3–11]	3 [3–10]	7 [3–11]	0.017*
ED GCS ≤ 8	389 (65)	216 (66)	173 (64)	0.139
Of whom sedated	303 (78)^c^	203 (94)^c^	100 (58)^c^	< 0.001*
Anisocoria/ unresponsive pupil	159 (27)	106 (33)	53 (20)	< 0.001*
Absent cornea reflex^b^	75 (13)	47 (14)	28 (10)	0.138
n/a (or sedated)	469 (79)	265 (81)	203 (75)	
Ethanol intoxication^c^	128 (21)	68 (21)	60 (22)	0.437
Anticoagulant use				0.743
None	450 (76)	244 (75)	206 (76)	
Antiplatelet therapy^d^	58 (10)	31 (10)	27 (10)	
Coumarins/heparins	61 (10)	33 (10)	28 (10)	
DOAC	4 (1)	1 (1)	3 (1)	
Radiologic findings^e^				
Epidural hemorrhage	150 (25)	73 (22)	77 (29)	0.088
Subdural hemorrhage	431 (72)	234 (72)	197 (73)	0.748
Subarachnoid hemorrhage	428 (72)	234 (72)	194 (72)	1.0
Compression basal cisterns	178 (30)	96 (29)	82 (30)	0.857
Midline shift ≥ 5 mm	215 (36)	118 (36)	97 (36)	1.0

Data are expressed in medians [Q1–Q3] or absolute numbers (%)

*UMC-R* University Medical Center Rotterdam; *UMC-U* University Medical Center Utrecht; *RTS* Revised Trauma Score; *ISS* Injury Severity Score; *AIS* Abbreviated Injury Scale; *HEMS* Helicopter Emergency Medical Service; *ED* Emergency Department; *DOAC* direct oral anti-coagulant; *GCS* Glasgow Coma Scale; *n/a* not applicable

*Statistically significant (*p* < 0.05)

^a^Index based on the RTS

^b^Only performed in patients with GCS = 3, reported as either one or both eyes

^c^Based on either clinical assessment or serum intoxication screening

^d^These patients solely used antiplatelet therapy

^e^Several patients had more than one radiological finding on CT head; initial CT head in the ED

### Clinical variables

Prehospital deployment of HEMS was notably and statistically more frequent in the UMC-R than UMC-U (66 vs 35%, respectively; *p* < 0.001). No statistical differences were measured in prehospital GCSs (prior to airway management and sedation) (*p* = 0.626) between both centers. UMC-R patients were statistically more often sedated in prehospital setting and Emergency Department (ED) than UMC-U patients (84 vs. 40%, respectively; *p* < 0.001). Subsequently, GCS in ED was significantly lower in UMC-R compared to UMC-U patients (3 [3–10] vs. 7 [3–11], respectively; *p* = 0.017). However, the number of patients with GCS ≤ 8 in ED was similar between both centers (UMC-R: 66% vs. UMC-U: 64%, *p* = 0.139). In addition, 94% of UMC-R patients with GCS ≤ 8 was sedated compared to 58% in UMC-U patients (*p* < 0.001, Table 1). Radiological findings were statistically comparable for all reassessed variables between centers as displayed in Table 2.

**Table 2:** Neurosurgical treatment and outcome variables

	Total *N* = 596	UMC-R *N* = 326	UMC-U *N* = 270	*p* value
Neurosurgical treatment				
ICP monitor	118 (20)	98 (30)	20 (7)	< 0.001*
Craniotomy	182 (31)	88 (27)	94 (35)	0.039*
Outcome				
ICU-LOS, days	4 [2–9]	4 [2–10]	4 [2–8]	0.295
H-LOS, days	12 [5–23]	11 [4–22]	13 [5–26]	0.165
In-hospital mortality	170 (29)	94 (29)	76 (28)	0.853
6 month mortality	180 (30)	100 (31)	80 (33)	0.783
WLST	123 (21)	60 (18)	63 (23)	0.139

Data are expressed in medians [Q1–Q3] or absolute numbers (%)

*Statistically significant (*p* < 0.05)

*UMC-R* University Medical Center Rotterdam; *UMC-U* University Medical Center Utrecht; *ICP* intracranial pressure; *WLST* withdrawal of life sustaining treatment; *ICU* Intensive Care Unit; *(H)-LOS* (hospital) length of stay

### Treatment and outcomes

The use of cranial decompressions was significantly higher in UMC-U compared to UMC-R (35 vs. 27%, *p* = 0.039), while UMC-R patients received an intracranial pressure monitor more frequently than UMC-U patients (30 vs. 7%, *p* < 0.001). Patients from both hospitals had similar ICU-LOS (*p* = 0.295), and hospital LOS (*p* = 0.165). Further, there was no difference in in-hospital mortality rate (*p* = 0.856) and WLST rate (*p* = 0.139) between both cohorts. All treatment and outcome variables are displayed in Table 2. Among the 47 patients (8%) in whom treatment was not withdrawn, the majority died after being declared brain dead (*n* = 29, 62%) followed by infectious complications (*n* = 9, 19%). Four patients (9%) died following failed cardiopulmonary resuscitation whereas five patients (11%) died following other admission-related complications.

### Functional outcomes

Total survival rate was 71% (*n* = 426). The largest group of survivors was discharged to a rehabilitation facility (*n* = 144, 34%) followed by home discharge (*n* = 135, 32%). Functional outcome scores during follow-up are presented in Table 3. In total, ten patients (2%) were discharged with UWS (GOS = 2), five patients (1%) persisted in this state both at 30 day and 6 months follow-up. Ten patients died during follow-up, one patient improved from UWS (GOS 2) to severe disability (GOS 3), and one with severe disability (GOS 3) deteriorated to UWS (GOS 2).

**Table 3:** Glasgow outcome scores during follow-up

	Death^d^	Persistent vegetative state	Severe disability	Moderate disability	Good recovery	Missing^e^
UMC-R (*n* = 232)						
First assessment at 30 days^a^	4 (2)	2 (1)	59 (25)	75 (32)	39 (17)	53 (23)
Second assessment at 3–6 months^b^	6 (3)	2 (1)	30 (13)	46 (19)	45 (19)	103 (44)
Lost to follow-up at second assesment^c^			13 (6)	26 (13)	14 (6)	
UMC-U (*n* = 194)						
First assessment at 30 days^a^	2 (1)	3 (2)	87 (45)	59 (30)	12 (6)	31 (16)
Second assessment at 3 to 6 months^b^	4 (2)	3 (2)	33 (17)	60 (28)	27 (13)	67 (35)
Lost to follow-up at second assesment^c^			19 (10)	17 (9)	3 (2)	
Total (*n* = 426)						
First assessment at 30 days^a^	6 (1)	5 (1)	146 (34)	134 (32)	51 (12)	84 (20)
Second assessment at 3–6 months^b^	10 (2)	5 (1)	63 (15)	106 (25)	72 (17)	170 (40)

Data are non-cumulative and are expressed in absolute frequencies (%)

*Three patients were missing during first assessment but were present during second assessment interval

*UMC-R* University Medical Center Rotterdam, *UMC-U* University Medical Center Utrecht, *FU* follow-up

^a^Patients had their first follow-up approximately between 4 and 6 weeks

^b^Approximately; secondary follow-up varied from 2 to 6 months

^c^Patients who were lost to follow-up are displayed according to their respective last known outcome score

^d^Includes only patients who died after discharge, displayed as cumulative

^e^Also includes patients directly lost to follow-up

## Discussion

This multicenter retrospective cohort study compared outcomes after moderate-to-severe TBI in two Dutch trauma populations admitted to a level-I trauma center. The in-hospital TBI-associated mortality rate in this study (29%) is on the low end compared with rates 28–36% reported in comparative literature [12, 28, 29]. Most patients died of TBI after WLST (72%) with statistically comparable rates in both centers.

The WLST rate of 72% (of total in-hospital mortality) in the present study corresponds with the few comparative studies on TBI-related WLST incidences ranging from 58 to 86%[12, 14, 29–32]. A brief comparison of relevant literature has been provided in Appendix Table 5. Contrarily, a survey among European clinicians on estimated WLST rates reported that in 60% of the participating centers, over 50% of the patients with severe neurological injury died after WLST, which may indicate a disparity in physician-reported measures and actual WLST rates [33]. Moreover, a literature review by Leblanc et al. on TBI—atients participating in randomized controlled trials rarely mention WLST rates [13]. The lack of structured WLST information in patient records and management reports is likely due to the sensitive nature of the subject which is not without controversy. An end-of-life decision as WLST in a patient—who has not been declared braindead—is a weighty and serious decision and requires due diligence. Errors in this process may result in legal ramifications. Such precedents may discourage hospitals from disclosing these figures for fear of exhibiting contempt of ethical practice or lack of quality of health care. In turn, this may result in condemnation from medical boards and society alike. For instance, in German neurosurgical treatment facilities, end-of-life decisions have given many occasions for controversies and clinicians treating neurological injuries are still unaccustomed to end-of-life practices [34, 35].

Patients in UMC-R scored higher AIS scores and lower GCSs in ED compared to UMC-U patients. However, Probability of Survival rates, radiological findings, prehospital GCS, and outcomes were all statistically comparable, while the use of prehospital sedation was significantly higher in UMC-R. These differences are likely due to the more frequent involvement of HEMS teams and subsequently more usage of sedation for airway management. This difference in HEMS involvement may be due to the differing geographic composition of the UMC-R region compared to the UMC-U region and the availability of a proprietary HEMS team in UMC-R region. The use of sedation consequently results in lower GCSs in ED, while injury and outcome characteristics were comparable. This may also affect AIS and ISS scores as coma is incorporated into the AIS classifications as a clinical parameter for injury severity. This results in systematically higher AIS and ISS [24]. Therefore, the disparity in sedation use reflects the difference in AIS severity assessment by AIS coders. This may be an important finding for AIS classification of TBI patients in future studies, as well as applicability of the AIS system for hospital use. Furthermore, inter-observer variability may also have influenced the difference in AIS scores to some extent. The post hoc analysis comparing reassessment of the initial AIS scores between coders measured ICC’s fair-to-moderate. Thus, a tendency of subjective over-estimation of injuries could be present. A previous multicenter study (also including data from UMC-U) confirmed this phenomenon in AIS head assessment and showed significant inter-center variability across international centers. Intra-center agreement across coders, however, was perfect [8]. These findings are in line with disparities found in the current sample and may indicate a need for refinement and uniformity in AIS assessment in TBI patients.

Differences in the use of invasive measures (i.e., ICP monitoring and decompressive surgery) were evident in both centers. The role of ICP monitoring in TBI is a subject of debate [36]. Some studies indicate that ICP monitoring provides a higher degree of pressure monitoring and improves mortality and treatment outcomes when reliable neurologic evaluation is unattainable [37]. Others demonstrate no significant impact on outcomes compared to control groups, while associated with higher complication and mortality rates, particularly among elderly patients [38]. Our data suggest that even between two centers in the same country, attitudes toward treatment modalities such as ICP monitoring and cranial decompressions differ notably. In addition to the disagreement in literature, this may indicate the lack of consensus on which invasive measures and acute management are most beneficial to TBI patients.

This study showed a low incidence of patients discharged with UWS. We suspect that these rates are in line with Dutch reluctance on persisting a life in a vegetative state [39]. In addition, data from our study showed that nearly half of decisions to WLST were within 24 h after trauma. The Neurocritical Society recommends a 72-h observation period to assess brain damage, as WLST executed too early may pose a risk for a self-fulfilling prophecy [40]. However, a study by McCredie et al. compared the delay in WLST decisions in patients with TBI and concluded a non-inferior relation between the delay in WLST and mortality rate, time to death, and ICU length of stay [41]. It is assumed that patients who received WLST within 24 h suffered from refractory injuries with a near-certain risk for UWS or death.

This study had several limitations. First, the study was limited due to the nature its retrospective design for collecting in-hospital and prehospital data. Consequently, the prehospital GCS was commonly performed by paramedics with presumable varying accuracy on their neurologic examinations. As this is a commonly used parameter for TBI, severity in literature comparing prehospital GCS or assessing prehospital TBI severity should be done with this possible bias in mind. Subsequently, the reported use of sedation and its possible effect on ISS and AIS scores—which in itself may be an important finding on the validity of the scoring systems—may also affect the accuracy of TBI severity when comparing to other studies. A second limitation was usage of GOS, used to correlate clinical parameters and assess neurologic recovery in patients with severe TBI after intensive therapy. An extended version of GOS has been developed over time which better integrates functional goals [42]. However, the newer version was not yet incorporated in both participating centers during the earlier part of the observed period. Lastly, the follow-up period had several missing patients, so their functional outcomes are unknown. However, mortality was accurately recorded from the Dutch national registry of deceased persons.

Decisions leading to WLST in TBI encompasses demanding situations. Despite the availability of various diagnostic modalities, registration and uniform (inter)national guidelines are lacking. This may be reflected by the formerly mentioned disparity in use of ICP-meters compared to decompressive craniotomies. In addition, variability among acute care clinicians concerning end-of-life treatment, neurologic prognosis, and end-of-life documentation remains high [33, 43, 44]. Guidelines can be useful for registration purposes, but also for providing ethical guidance, and avoiding self-fulfilling prophecies in a practice more or less based on expert opinions [43, 45].

This study adds to the presumption that WLST after TBI is of common use in the Netherlands despite the few comparative studies. Besides addressing possible associated effects regarding TBI mortality or end-of-life decisions, future research should explore the effect of either ICP-monitors or decompressive surgery on mortality and WLST rates, as different frequencies were observed in this study. In addition, this study may indicate a need to revalidate the use of the AIS and the use of GCS after TBI, as there were notable differences in sedation use and AIS scores while clinical and prehospital parameters were statistically comparable. Furthermore, the appropriateness and potential self-fulfilling effects of WLST warrant further investigation. A comparative observational study of trauma centers in different countries with high versus low rates of WLST, supplemented with prehospital vital signs, clinical and radiological assessments, and end-of-life decisions, could provide valuable insights on this matter. Such a study could also examine the cultural and ethical factors that influence WLST practices across different countries.

This multicenter study of two Dutch level-I trauma centers reported comparable outcomes in isolated TBI patients wherein most deaths resulted from WLST (72%) and merely 2% were discharged with UWS. This may indicate that WLST is common in the Dutch setting for moderate-to-severe isolated TBI. Results also demonstrated a difference in invasive measures between centers and a possible effect of prehospital sedation on the AIS and ISS scores for TBI severity assessment. This study also suggests that WLST may be a key contributor to the global rise of TBI-related mortality compared to other traumatic causes.

## Data Availability

The data supporting this article are available via the corresponding author upon reasonable request.

## Appendix 1

See Tables 4, 5.

**Table 4:** Guidelines for agreement measures [22]

0.00–0.20	Slight
0.20–0.40	Fair
0.40–0.60	Moderate
0.61–0.80	Substantial
0.81–1.00	Almost perfect

**Table 5:** Comparison of literature reporting WLST rates in TBI

Study	Turgeon et al.	Van Veen et al.	Tanizaki et al.	Gambhir et al.	Izzy et al.	Tran et al.	Present study
Study specifications							
Design	Retrospective cohort	Prospective cohort	Retrospective cohort	Retrospective cohort	Retrospective cohort	Retrospective cohort	Retrospective cohort
Centers	Six centers, Canada	63 centers, 17 European countries and Israel	Single-center, Japan	National database, USA	Single-center, USA	Single-center, Canada	Two centers, the Netherlands
Year published	2011	2021	2023	2020	2013	2023	2023
Observed period	2005–2006	2014–2017	2012–2021	2010–2016	2009–2013	2014–2015	2011–2016
Cohort size	720	2022	161	328,588	232	126	596
Inclusions	Mechanically ventilated, severe TBI, age ≥ 16 years	Clinical TBI diagnosis, ICU admission, age ≥ 16 years	Severe TBI, age ≥ 18	Severe TBI, age ≥ 18	Moderate–severe TBI; post-resuscitation GCS ≤ 12,	Isolated TBI, ISS > 12, activation of trauma team, age ≥ 16 years	Moderate–severe isolated TBI, ICU admission
Exclusions	Penetrating injuries	n/s	Cardiopulmonary arrest on arrival	n/s	Absence of a non-traumatic brain lesion on CT, hanging, drowning	Transferred > 48 h, low GCS unrelated to TBI	Hospital transfers, isolated spine injuries
TBI definition and severity assessment	GCS ≤ 8 on admission	Clinical diagnosis and need for CT brain	AIS head 3–5 and GCS 3–8 on arrival	AIS head 3–5	GCS ≤ 12	AIS head ≥ 3, AIS other region ≤ 2	AIS head ≥ 3, AIS other region ≤ 2
Demographics and outcome							
Age	42,4 (± 20,5)	n/s	n/s	n/s	53^a^	67 (Q1-Q3: 44–80)	57 [Q1-Q3: 38–70]
Age—WLST patients	53,9 (± 21,2)	60 [Q1-Q3: 39–74]	77 [Q1-Q3: 67–84]	61 (IQR: 38)	64 (± 21)	n/s	68 [Q1-Q3: 54–78)
Sex, male, *n* (%)	555 (77)	n/s	109 (68)	216,955 (66)	161 (69)	82 (65)	379 (64)
In-hospital mortality, *n* (%)	228 (32)	267 (13)	87 (54)	n/s	84 (36)	69 (55)	170 (29)
WLST, *n* (% of mortality)	160 (70)	229 (86)	52 (58)	18,257 (6)^b^	57 (68)	50 (72)^c^	123 (72)

*USA* United states of America; *TBI* traumatic brain injury; *ICU* Intensive Care Unit; *GCS* Glasgow Coma Score; *ISS* Injury Severity Score; *CT* computed tomography; *AIS* Abbreviated Injury Scale; *WLST* withdrawal of life sustaining treatment; ± : standard deviation; Q1-Q3: quartile 1–quartile 3; IQR: interquartile range; n/s: not/none specified

^a^No standard deviation or interquartile range provided

^b^No in-hospital mortality rate provided, percentage of total cohort

^c^Includes only WLST < 1 week post-injury
